# From chaos to control: Nobel insights in regulatory T cells and immune tolerance

**DOI:** 10.1007/s00424-025-03140-1

**Published:** 2025-12-10

**Authors:** Anna Estrada Brull, Nicole Joller

**Affiliations:** Department of Quantitative Biomedicine, University of Zurich, Zurich, Switzerland

## Abstract

The 2025 Nobel prize in Medicine or Physiology was awarded to Mary E. Brunkow, Fred Ramsdell and Shimon Sakaguchi for unravelling the basis of peripheral immune tolerance: regulatory T (Treg) cells. Treg cells are a subset of CD4^+^ T helper cells characterized by the expression of the transcription factor *Foxp3* and the surface receptor CD25 (IL-2 receptor alpha subunit) and are key for maintaining immune homeostasis and preventing autoimmunity. In this minireview, we trace the discovery of Treg cells, their phenotype and function and how these findings are employed in generating safer and more efficient therapies for autoimmune diseases and cancer.

## Introduction

The immune system fulfills a fundamental task: protecting the body from any form of harm, mainly external (infections) but also internal (aberrant cells, cancer). But how does it achieve this protection in an efficient manner? To meet this challenge, the immune system evolved the adaptive immune branch, which, once activated, mounts specific and long-lasting responses against the pathogens. The adaptive immune system is composed of T cells, which coordinate the immune response and kill infected cells, and B cells, which produce antibodies that specifically recognize and neutralize antigens.

T cells express a unique T cell receptor (TCR) which recognizes antigens presented on major histocompatibility complex (MHC) molecules. There are two types of MHC molecules: class I, expressed by all nucleated cells, presents peptides derived from intracellular proteins; and class II, expressed primarily by professional antigen-presenting cells (APCs), presents peptides originating from the extracellular environment. Each T cell expresses several copies of a single, clonally unique TCR, allowing it to recognize only one specific peptide-MHC complex. CD8^+^ T cells recognize intracellular antigens bound in MHC class I and are responsible for eliminating infected or abnormal cells. CD4^+^ T cells recognize peptides presented on MHC class II and coordinate the activity of other immune cells by secreting cytokines.

Providing specificity for all kinds of pathogens is achieved thanks to a wide TCR diversity. T cell precursors have the potential to generate up to 10^18^ different TCR combinations through random gene rearrangement. However, high TCR diversity increases the risk of having non-functional or self-reactive T cells and requires a selection process to ensure that only functional, non-pathogenic T cells patrol the body. Both the TCR generation and selection, as well as the final expression of the CD4 or CD8 co-receptors occur during T cell maturation in the thymus. T cell precursors migrate from the bone marrow and seed the thymus, where they undergo proliferation and differentiation. Thymic epithelial cells express MHC class I and class II molecules and are capable of presenting many different self-peptide combinations. Binding to a peptide-MHC combination with intermediate levels will provide survival and maturation signals to the developing T cell and will determine whether they become CD4^+^ or CD8^+^ T cells. Developing T cells with TCR rearrangements that are not capable to bind to MHC molecules in the thymus die of neglect. On the other hand, those that have high affinity for peptides of the host would cause autoimmune responses and are therefore eliminated by apoptosis before leaving the thymus, a process called negative selection and the basis of central tolerance [1].

In the human body, an estimated 10^11^ T cells circulate with a TCR repertoire that consist of 10^6^ to 10^8^ unique sequences [1]. Considering the enormous task of thymic selection and the fact that most biological processes are imperfect, what happens if some clones escape negative selection and get activated in the peripheral tissues? Are there other mechanisms keeping them in check?

## The discovery of Treg cells

Researchers studying the thymus realized that postnatal thymectomy drove several autoimmune disorders [2]. This brought the idea that some T cells could act as suppressors of autoimmunity. The Nobel laureate Shimon Sakaguchi and colleagues observed that thymectomized mice had decreased numbers of CD5^hi^ CD4^+^ T cells, and that transfer of this population could prevent the development of autoimmunity [3]. Hence, these “suppressor cells” appeared to be enriched among CD5^+^ cells. On the other hand, reports highlighted that transfer of CD45RB^hi^ cells induced severe wasting disease, whereas concomitant transfer of CD45RB^lo^ cells could prevent it [4]. In 1995, Sakaguchi looked for a surface marker that would be present on these ‘suppressor T cells’ by looking for proteins showing positive correlations with CD5 and inverse correlations with CD45RB. This approach identified CD25 (Interleukin-2 receptor alpha chain, IL-2Rα), which was expressed on approximately 10% of CD4^+^ T cells [5]. By depleting the CD25^+^ CD4^+^ population (now known as Treg cells) and transferring the remaining T cells to athymic mice, they could observe the development of autoimmunity. In contrast, co-transfer of these CD25^+^ CD4^+^ Treg cells could reduce or even prevent autoimmunity [5].

In 2001, Mary Brunkow and Fred Ramsell were working on elucidating the cause of the phenotype of a mutant mouse called “Scurfy”^6^. Male animals from the scurfy strain had scaly skin, ruffled fur and, most importantly, suffered from fatal autoimmunity. By mapping the disease relevant area of the X chromosome through shotgun sequencing, they identified 20 putative genes. Among those, only one showed a change in the protein-coding sequence in scurfy mice. The gene, named *Foxp3*, encoded for a winged-helix domain protein with homology to the forkhead family and was a transcriptional regulator. To validate that the mutation in *Foxp3* was causing the scurfy phenotype, they performed complementation studies where transgenic mice were generated by injecting functional copies of the *Foxp3* gene into mouse oocytes. Scurfy males carrying the transgene had normal lymph nodes and survived past weaning, confirming the mutation in *Foxp3* as the reason for the disease [6].

Around the same time, a severe autoimmune disorder later called Immunodysregulation Polyendocrinopathy Enteropathy X-linked (IPEX) syndrome was described [7, 8]. Ramsell, Brunkow and colleagues saw the similarity between the symptoms of IPEX patients and scurfy mice [9]. After sequencing different families affected with IPEX, they could determine that the cause were again mutations in the *FOXP3* gene [10, 11].

**Fig. 1:**
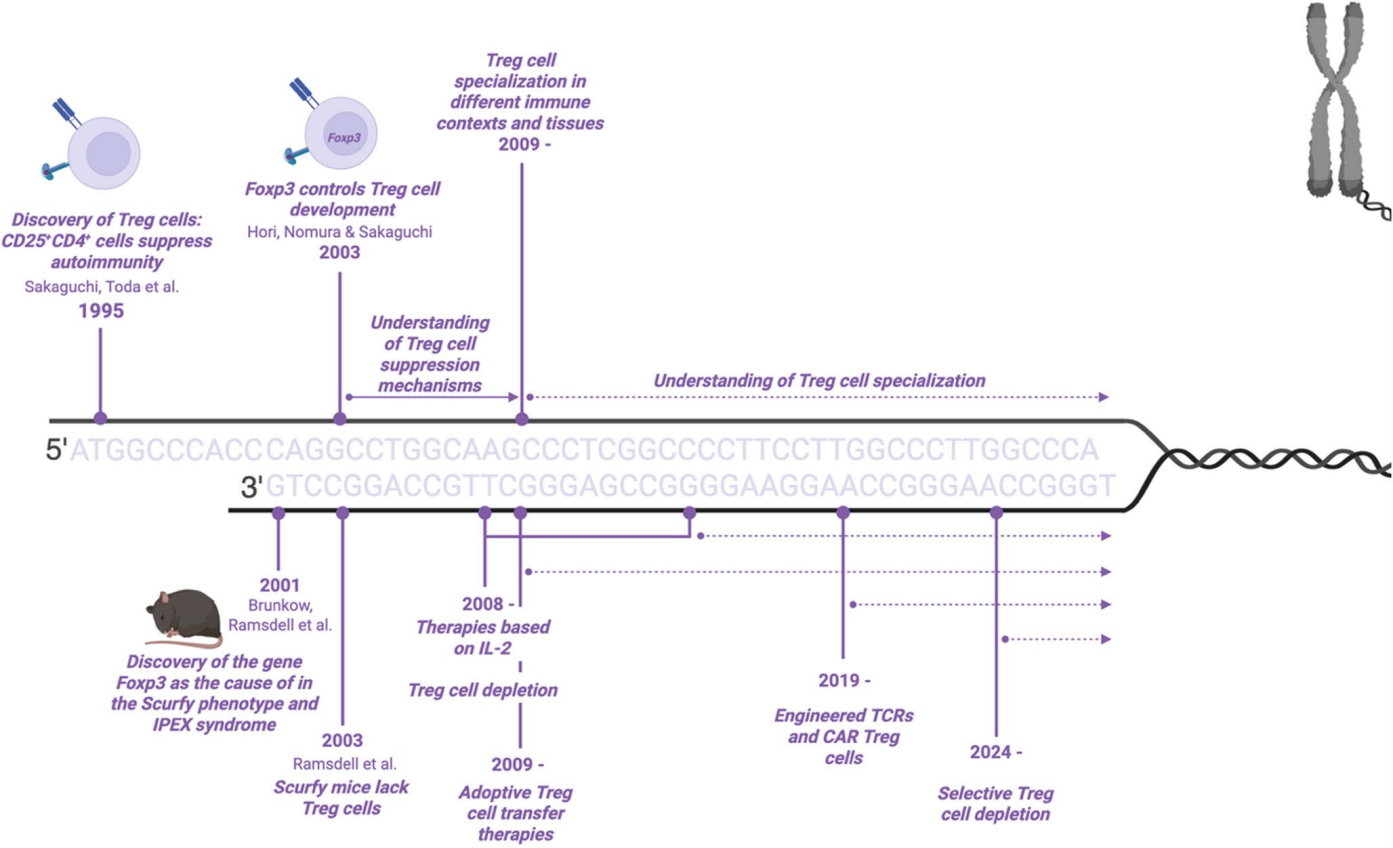
Timeline of major breakthroughs in Treg cell research. Timeline describing the first Treg cell discoveries as well as points when new concepts or novel therapies were applied. For therapeutic strategies, dates corresponding to their first application in humans are indicated. Figure created with Biorender.com

When characterizing *Foxp3*, Brunkow and colleagues found it to be most highly expressed in lymph nodes, thymus and spleen; specifically in CD4^+^ CD8^−^ T cells. They also described that *Foxp3* expression was required for T cell function and could limit their activation [6]. Soon after, Sakaguchi’s group determined that *Foxp3* was predominantly expressed in the suppressive CD25^+^ CD4^+^ T cells, identifying *Foxp3* as the master transcription factor for Treg cells [12]. Expression of *Foxp3* was stable and independent of the activation status of the cells and it conferred T cells with suppressive capacity in vitro and in vivo. Simultaneously, a team lead by Ramsdell proved that scurfy mice lacked Treg cells [13] (Fig. 1).

## Function of Treg cells

There is a fine-tuned equilibrium between Foxp3^−^ T cells (called effector T cells) and Treg cells which contributes to maintaining homeostasis. A key cytokine involved in Treg cell survival is IL-2. Activated T cells produce IL-2, which enables them to undergo clonal expansion and thereby, effective T cell responses. In contrast, Treg cells cannot produce IL-2 but rely on external IL-2 for their survival [14]. As Treg cells express the high affinity receptor for IL-2, formed by the addition of CD25 into the low affinity IL-2 receptor complex, they preferentially consume the IL-2 produced by the activated T cells. This mechanism not only allows for Tregs to survive and proliferate but also to dampen effector T cells expansion [15, 16], ensuring that Treg cells and effector T cells remain in a dynamic equilibrium.

Besides consuming IL-2, Treg cells can deprive other immune cells from exogenous ATP through the expression of the ectoenzymes CD39 and CD73 on their surface, reducing overall cell activation and inflammation. Alternatively, Treg cells can also block proliferation and differentiation of other lymphocytes by direct transfer of cyclic adenosine monophosphate (cAMP) via gap junctions, which can in turn inhibit cytokine transcription in the target cells [17].

Other mechanisms involving contact-dependent suppression include co-inhibitory receptors. Treg cells express high levels of the co-inhibitory receptor CTLA-4, which can compete for binding to CD80 and CD86 and deprive effector T cells from these costimulatory cues required for proper activation. Other co-inhibitory receptors such as LAG-3, TIM-3, TIGIT or CD85k are upregulated in Treg cells upon activation or in certain inflammatory contexts, where they also contribute to the suppressive function of Treg cells [18–20].

**Fig. 2:**
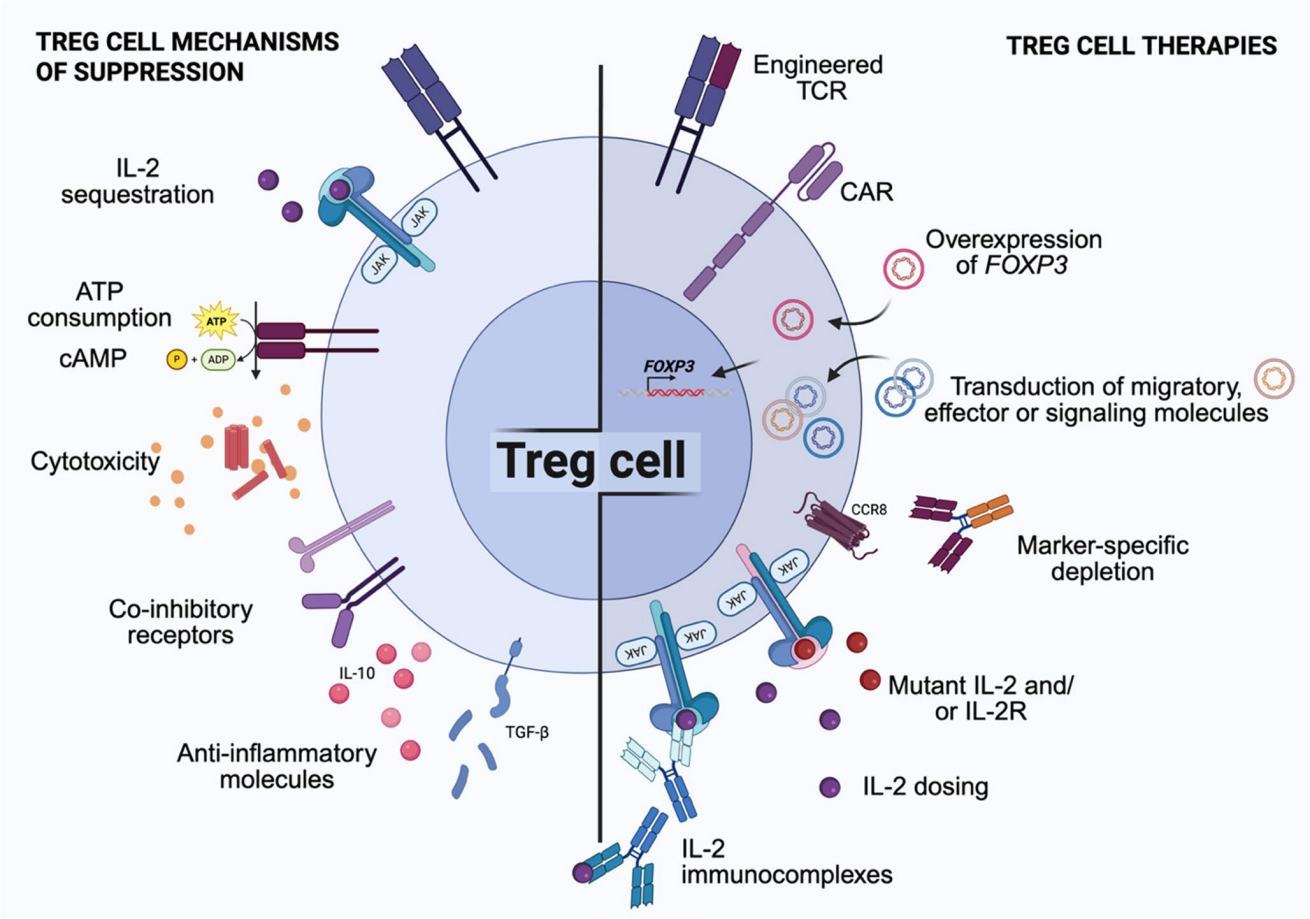
Treg cell mechanisms of suppression and targets for therapies. (left) Treg cells suppress other immune cells by consuming IL-2 and ATP, transferring cAMP and cytotoxic molecules, releasing anti-inflammatory mediators and through direct contact via co-inhibitory receptors. **(**right**)** Strategies for immune modulation targeting Treg cells. Enhancing Treg cell function can be achieved with engineered antigen-specific TCR, CARs, overexpression of *FOXP3* or other desired proteins, or modulating the IL-2 pathway. Treg cells can also be compromised by diverting IL-2 signaling or depleted through targeted antibodies. Figure created with Biorender.com

Finally, Treg cells can also produce immunosuppressive cytokines, like IL-10 and TGF-β or cytotoxic molecules to dampen or kill target cells, respectively [17]. The main Treg cell suppressive mechanisms are illustrated in Fig. 2 (left).

## Treg cell specialization: a new beginning

In 2009, Koch and colleagues discovered that, like Th1 cells, Treg cells can also express the transcription factor T-bet [21]. In fact, transferring a pool of T-bet KO Treg cells into scurfy mice could not prevent the development of autoimmunity [21]. This finding opened a new door for Treg cell research, as it suggested that Treg cells can specialize into different subsets to control certain branches of the immune response without compromising general immune tolerance and homeostasis.

As it became clear that Tregs cells not only express FOXP3 but, depending on the context, also co-express other transcription factors, it was further noted that Treg cells can seed peripheral tissues and perform non-canonical functions adapted to the tissue they reside in. At barrier sites, Treg cells can promote epithelium regeneration and control tissue-specific cell expansion and mucus production. Treg cells can also interact with stem cells and promote their expansion upon injury, counteract fibrosis in skeletal and cardiac muscle, promote remyelination and enhance hair growth. While some of these functions are associated with the reduction of inflammation and the switch in macrophages towards an anti-inflammatory phenotype, part of these rely on the direct interaction of Treg cells with the tissue and their production of growth factors [22].

In addition to growth or transcription factors, other markers such as co-inhibitory receptors or chemokine receptors are also used to segregate Treg cell subsets. For example, TIGIT^+^ Treg cells play a key role in repairing collateral damage caused by pathogen clearance and anti-infectious immune responses [23] and CD85k expression on Treg cells allows for suppression of activated T cells during persistent viral infections [19]. As such, many other Treg cell markers have been described in different contexts [18, 24], and could be exploited to specifically target Treg cells for precise immune control as opposed to global immunosuppression.

## Therapies targeting Treg cells

While Treg cells play a beneficial role in preventing autoimmune reactions, they are detrimental in chronic infections and cancer where they counteract responses aimed at eliminating pathogens or tumor cells. Thus, the possibility to modulate Treg cell function could have an impact in a wide range of diseases. Three decades after their first discovery, the understanding of Treg cells we have gained can now be exploited for targeted therapies (Fig. 1).

Generally speaking, Treg cell therapies can be grouped into three main approaches (Fig. 2, right):

### Adoptive Treg cell transfer

Transferring Treg cells after expansion was proven to be effective in preclinical models of several autoimmune diseases [25]. While polyclonal Treg cell transfer has been successful, transfer of antigen-specific Treg cells provides better suppression, reducing the number of Tregs that need to be transferred. Thus, novel therapies ensure specificity for a certain disease or tissue either through TCR engineering or Chimeric Antigen Receptor (CAR) expression. Engineered Treg cells show better suppressive capacities and improved migration to the inflamed tissues [26].

CAR-Treg cells are engineered to express the intracellular and costimulatory tails of a TCR receptor fused with the antigen binding site of an immunoglobulin at the extracellular site. CAR-Treg cells can recognize full proteins rather than presented peptides and are not restricted to MHC molecules, which broadens both the range of target antigens as well as the potential patient cohort.

Similar to the modifications in specificity achieved through CARs or transgenic TCRs, Treg cell migration or suppressive mechanisms can be enhanced by directly inserting the genes of interest (such as chemokine receptors or suppressor molecules) before expansion, or alternatively, by using culture and expansion conditions that favor their expression [26].

As Treg cells only comprise 5–8% of the total CD4^+^ T cell population, other groups are working on the conversion of effector CD4^+^ T cells into Tregs by culturing them in Treg-generating environments and/or by overexpressing *Foxp3*^26^. Nevertheless, probably due to the epigenetic imprints required for stable Tregs, the potential of these cells to fall back to their effector role and worsen the disease is higher than with *bona fide* Treg cells, entailing a risk that needs to be cautiously addressed.

### In vivo Treg cell expansion

As previously mentioned, IL-2 preferentially binds CD25 on Treg cells. Hence, low-dose IL-2 treatment drives Treg but not effector T cell expansion in vivo. Other strategies have been developed, such as mutant IL-2 molecules and receptors or IL-2–antibody complexes that redirect IL-2 specifically to Treg cells [26]. By favorizing Treg cell expansion, these therapies allow for better immune control and reduced inflammatory responses.

### Therapies depleting or hindering Treg cells

In conditions where Treg cells are detrimental (e.g. cancer), enhancing effector responses by suppressing Treg cells or modulating the IL-2 pathway can be employed. As opposed to low-dose IL-2 treatment, high doses of IL-2 preferentially enhance the effector T cell response. In addition, IL-2–antibody complexes can also redirect IL-2 to effector T cells instead of Treg cells. These methods shift the balance towards greater activation and expansion of effector T cells.

In addition to these approaches, direct Treg cell depletion has also been tested. Targeting Treg cells for antibody-dependent cell cytotoxicity with anti-CTLA-4, which preferentially binds Treg cells, was one of the first strategies to deplete Treg cells in tumors. However, with this approach Treg cell depletion was so efficient that it was often accompanied by autoimmune-like adverse effects. As a result, current efforts are focused on selectively depleting specific Treg cell subsets via antibodies against subset-specific markers, with the goal of minimizing systemic immune suppression and reducing associated side-effects [24, 27].

## Conclusions

Many of these therapies are being explored in clinical trials for a wide range of autoimmune diseases and cancer settings. Thanks to the discovery of Treg cells by the Nobel laureates, a big field of research in immune regulation began. The high number of clinical trials that directly or indirectly target Treg cells highlights the importance of these cells and their discovery. Thus, it is fitting that what started as the identification and characterization of CD25^+^ T cells was recognized with the Nobel Prize in Medicine or Physiology in 2025. Because in times of chaos, the best assets are not those that fight, but the ones that maintain the peace.

## Data Availability

No datasets were generated or analysed during the current study.
